# Facile Synthesis of Hierarchical Tin Oxide Nanoflowers with Ultra-High Methanol Gas Sensing at Low Working Temperature

**DOI:** 10.1186/s11671-019-2911-4

**Published:** 2019-03-08

**Authors:** Liming Song, Anatolii Lukianov, Denys Butenko, Haibo Li, Junkai Zhang, Ming Feng, Liying Liu, Duo Chen, N. I. Klyui

**Affiliations:** 1 College of Physics, State Key Laboratory of Superhard Materials, Jilin University, Changchun, 130012 People’s Republic of China; 2 V. Lashkaryov Institute of Semiconductor Physics, National Academy of Sciences of Ukraine, 41 Prospect Nauki, Kyiv, 03028 Ukraine; 3 Key Laboratory of Functional Materials Physics and Chemistry of the Ministry of Education, Jilin Normal University, Siping, 136000 China

**Keywords:** Hierarchical tin oxide nanoflowers, Hydrothermal method, Gas sensor, Methanol

## Abstract

**Abstract:**

In this work, the hierarchical tin oxide nanoflowers have been successfully synthesized via a simple hydrothermal method followed by calcination. The as-obtained samples were investigated as a kind of gas sensing material candidate for methanol. A series of examinations has been performed to explore the structure, morphology, element composition, and gas sensing performance of as-synthesized product. The hierarchical tin oxide nanoflowers exhibit sensitivity to 100 ppm methanol and the response is 58, which is ascribed to the hierarchical structure. The response and recovery time are 4 s and 8 s, respectively. Moreover, the as-prepared sensor has a low working temperature of 200 °C which is lower than that for other gas sensors of such type has been reported elsewhere. The excellent sensitivity of the sensor is caused by its complex phase mixture of SnO, SnO_2_, Sn_2_O_3_, and Sn_6_O_4_ revealed by XRD analysis. The proposed hierarchical tin oxide nanoflowers gas sensing material is promising for development of methanol gas sensor.

**Graphical Abstract:**

The as-obtained hierarchical tin oxide nanoflower (HTONF) gas sensor shows excellent gas-sensing performance at low working temperature (200 °C) and high annealing temperature (400 °C).
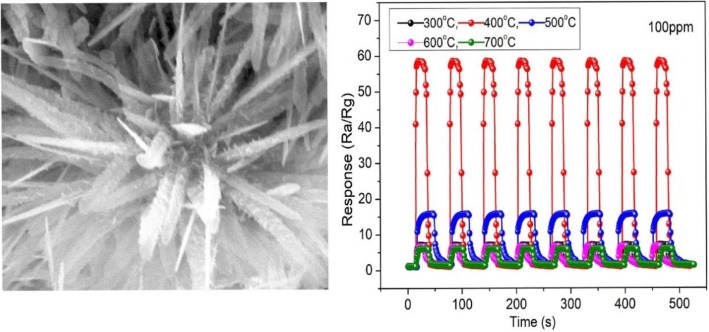

## Introduction

With the development of science and technology, methanol gas sensors have been widely applied in chemical industry. As a kind of colorless gas with a smell of ethanol, methanol can cause extremely serious health problems and environmental safety problems. For example, the central nervous system disorder and explosion would happen when the methanol concentration exceeds a threshold value.

Recently, the methanol gas sensing performance of variety of sensitive materials has been researched by a lot of researchers. For instance, Das et al. studied the gas sensing properties of the Mg^+ 2^:LaNiO_3_ thin film and obtained the response of 200–400 ppm methanol at 325 °C [1]. Ji et al. investigated the high-performance methanol sensor based on GaN nanostructures grown on a silicon nanoporous pillar array and gained the high gas sensing performance at the operating temperature of 350 °C [2]. Wang et al. reported methanol sensing properties of honeycomb-like SnO_2_ grown on a silicon nanoporous pillar array and showed the quick response properties at the operating temperature of 320 °C [3]. Although the above scientists have made an excellent progress, the high working temperature and cost sufficiently restrict the application of sensor, which motivates us to fabricate a high methanol performance gas sensor working at low temperature and prepared via a simple and low-cost synthetic method.

SnO_2_ gas sensors attract attention of researchers due to their simple and low-cost synthesis method and room temperature operation. The huge improvement of gas sensing performance has been achieved by decreasing crystallite size [4, 5], doping by metal elements [6, 7], fabricating heterostructure of metal oxide [8, 9], and, especially, fabricating hierarchical structure [10, 11]. To date, a variety of gas sensors has been proposed to improve gas sensing to methanol, such as In_2_O_3_ porous nanospheres [12], three-dimensionally macroporous LaFeO_3_ [13], and aluminum-mesoporous silicon coplanar [14]. However, the gas sensing properties of most of methanol gas sensors are not enough satisfactory. Therefore, the development of a kind of methanol gas sensor with high sensing properties at low working temperature by a simple method is still relevant.

In this work, we have tried to fabricate hierarchical tin oxide nanoflowers (HTONF) gas sensor to improve the gas sensing properties of sensor. We have performed a series of gas sensing examination of HTONF. And the results indicate the HTONF own excellent gas sensing performance (high sensitivity, good selectivity, rapid response and recover rates, and long stability) to methanol at low working temperature. The high gas sensing performance is caused by the hierarchical structure of the material and its phase composition, and this kind of hierarchical structure could provide many effective sites, which make the detected gas and material contact very well and extremely improve the gas sensing performance of the as-obtained materials.

## Methods

### Sample Preparation

**Fig. 1:**
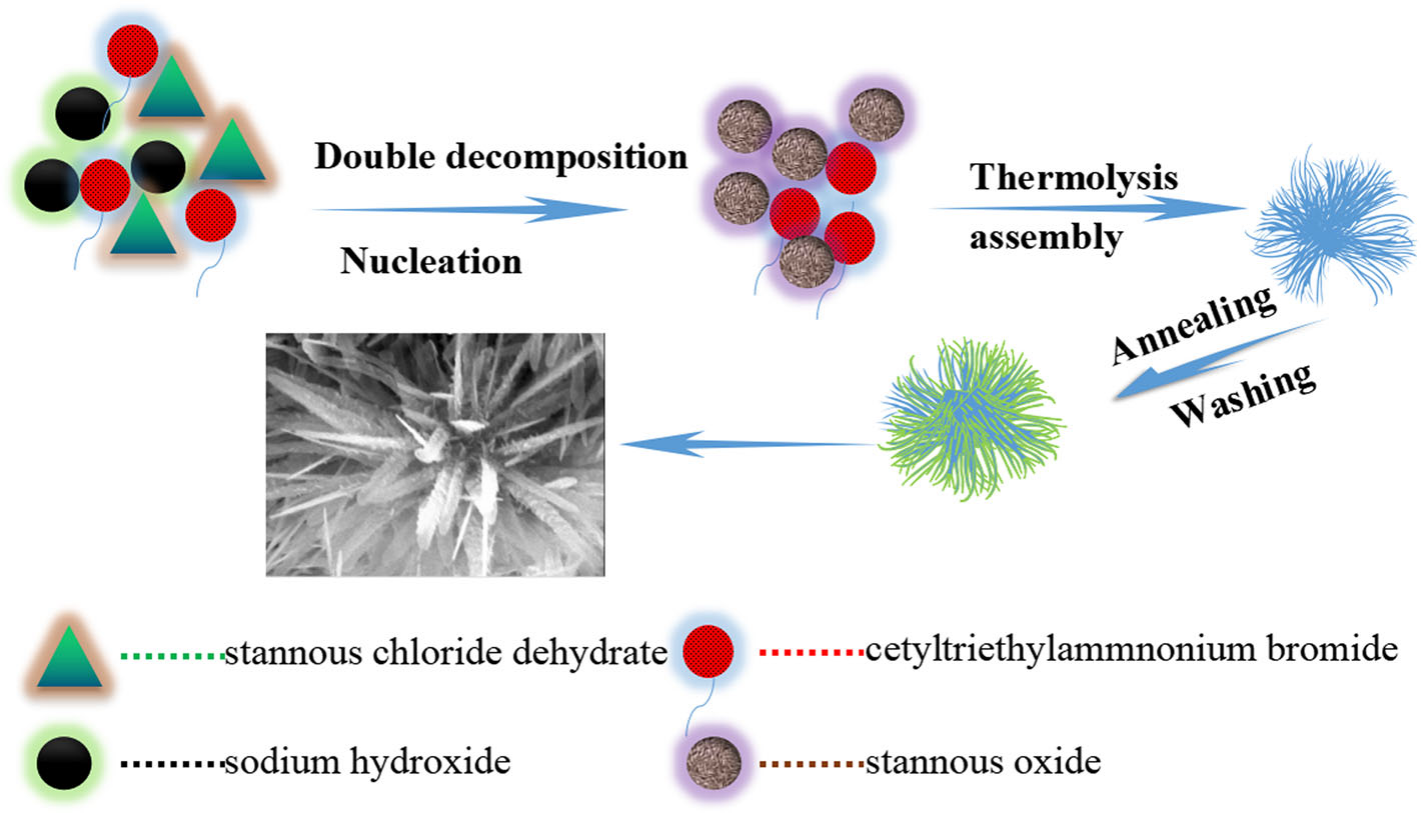
Schematic illustration of growth mechanism to prepare HTONF

Stannous chloride dehydrate (SnCl_2_·2H_2_O, 99.9%) and metyltrimethyl ammonium bromide (CTAB, 99.9%) were purchased from Sigma-Aldrich (USA). Sodium hydroxide (NaOH, 99.9%) was bought from Aladdin (China). The above chemical regents were analytical grade and were used without further purification. HTONFs were synthesized via the hydrothermal method. In brief, the solution of 2.2170 g stannous chloride dehydrate, 1.6032 g sodium hydroxide, and 0.7290 g metyltrimethyl ammonium bromide was dissolved and stirred in 35 ml distill water for 3 h. The as-obtained solution was transferred into a 50 ml Teflon-lined autoclave and heated for 5 h at 180 °C in a furnace and cooled down naturally. Then the resulting products were washed with distilled water and dried at 80 °C in a vacuum for 1 h. Finally, the as-obtained products were calcinated in a muffle furnace at 300, 400, 500, 600, and 700 °C for 3 h, and, as a result, the HTONF have been synthesized. The route of synthesis of HTONF is shown in Fig. 1.

### Fabrication of Sensor

**Fig. 2:**
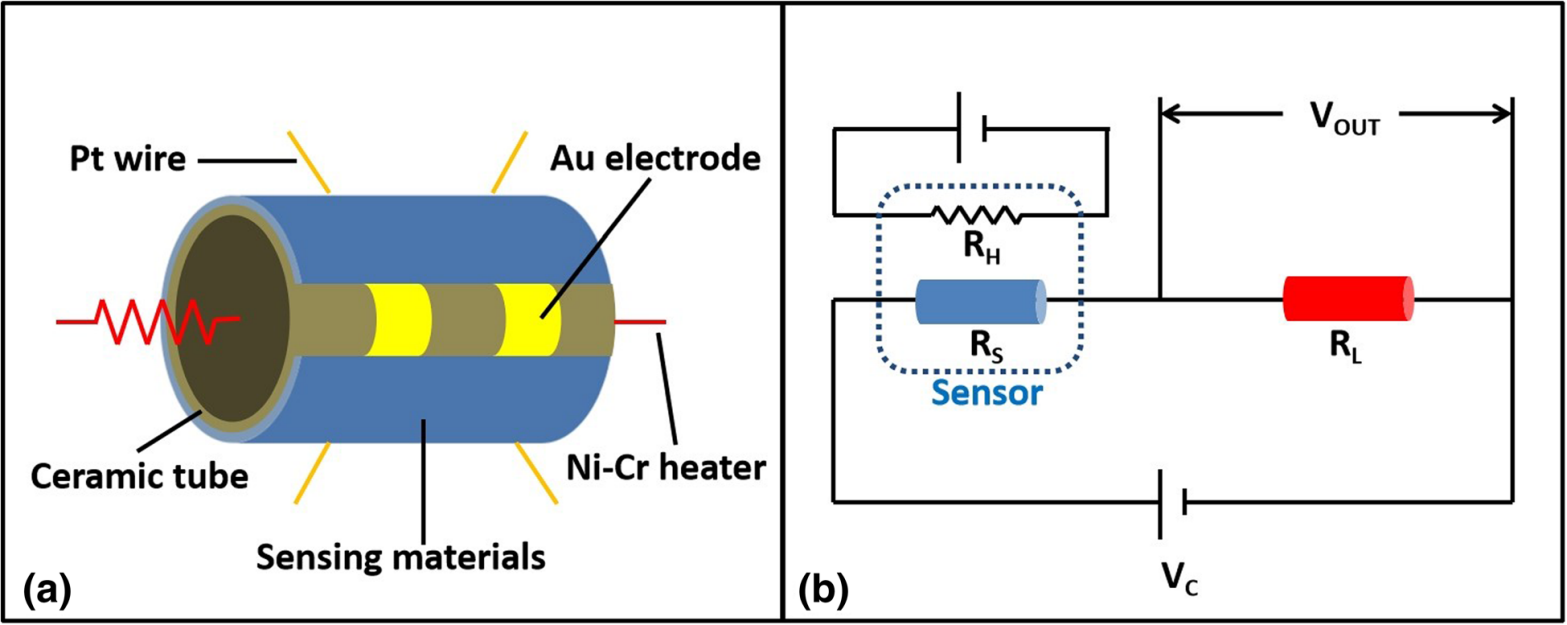
**a** Typical structure model of the gas sensor. **b** Schematic diagram of the circuit under test

The HTONF gas sensor was fabricated as follows: firstly, the powders were mixed with some amount of distilled water to form a paste. After that, the paste was printed uniformly on a ceramic tube with a pair of gold electrodes. Then, a Ni-Cr alloy wire coil, as a kind of heater, was inserted into a ceramic tube to control the operating temperature. The sensor response was defined as a ratio *R*_*a*_ to *R*_*g*_ (*R*_*a*_/*R*_*g*_), where *R*_*a*_ and *R*_*g*_ are resistances of structures in air and target gas, respectively [15, 16]. The schematic structure of the gas sensor device is presented in Fig. 2.

### Material Characterization

The crystallinity and structure of HTONF were characterized by X-ray diffraction (XRD) ADX-2700D X-ray Powder Diffraction Instrument with Cu_Kα_ radiation (λ = 1.15406 Å). The morphology of the samples was observed by scanning electron microscopy (FEI SKLSLM Magellan 400) and transmission electron microscopy (TEM, JEOLJEM-200FS). The specific surface area and pore size distribution of the samples were evaluated by Brunauer-Emmett-Teller (BET) model by Beijing JW-BK132F equipment. The mass changes of as-obtained HTONF powders were measured by thermal gravimetric analysis (TGA, SDT Q600, TA Instruments, USA). The CGS-8 system (chemical gas sensing, Beijing Elite Tech. Co. Ltd., China) was used to measure the gas sensing performance of samples.

## Results and Discussion

### Thermogravimetric Analysis

**Fig. 3:**
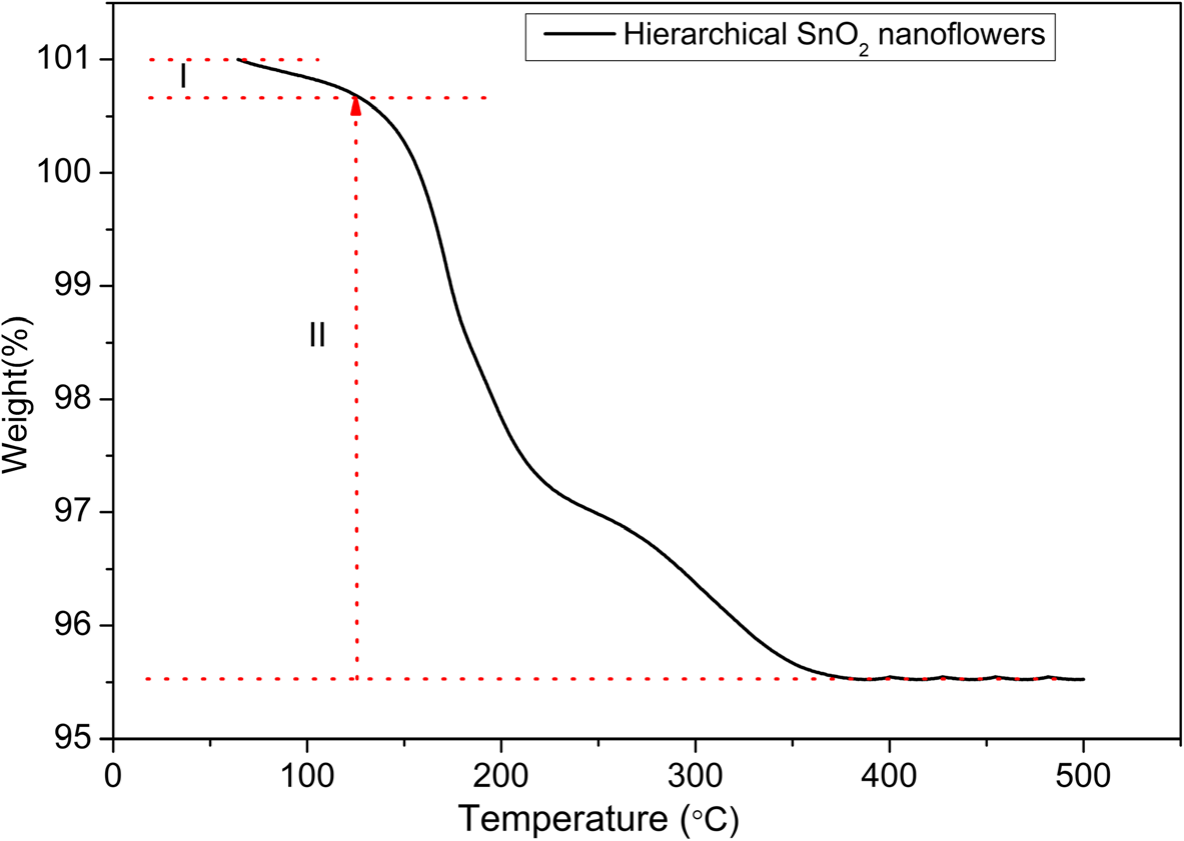
Thermogravimetric analysis curve of HTONF annealed in air atmosphere (heating rate of 5 °C/min from 30 °C to 500 °C in air)

The thermogravimetric analysis (TGA) curve of initial as-prepared powders is shown in Fig. 3; there are three stages of weight loss that can be distinguished. The first weight loss from 50 to 125 °C is due to the steaming of physiosorbed water molecule. The second weight loss in the range of 125–220 °C is attributed to the thermal degradation or the transformation of structure [17, 18]. The third stage lies in the temperature range 220–380 °С. And the thermogravimetric curve remains almost stable for temperatures over 400 °C.

### XRD and BET Analysis

It is well known that interaction of Sn (II) salts with alkaline solutions such as NaOH leads to formation of SnO_x_H_2_O (x < 1) [19]. So, in our case, the primary interaction of SnCl_2_ should correspond to the scheme:

1 $$ 6\ \mathrm{SnC}{\mathrm{l}}_2+12\ \mathrm{NaOH}\to \mathrm{S}{\mathrm{n}}_6{\mathrm{O}}_4{\left(\mathrm{OH}\right)}_4+12\ \mathrm{NaCl}+2\ {\mathrm{H}}_2\mathrm{O} $$

Dehydration of Sn_6_O_4_(OH)_4_ leads to formation of SnO which is gradually oxidized by oxygen of air to SnO_2_:

2 $$ \mathrm{S}{\mathrm{n}}_6{\mathrm{O}}_4{\left(\mathrm{OH}\right)}_4\to 6\ \mathrm{SnO}+2\ {\mathrm{H}}_2\mathrm{O}\uparrow $$

3 $$ 2\ \mathrm{SnO}+{\mathrm{O}}_2\to \mathrm{Sn}{\mathrm{O}}_2 $$

At the same time, the mechanism of transformation of SnO to SnO_2_ (Scheme 3) is quite complex and accompanied by appearing of different metastable crystalline phases (Sn_2_O_3_, Sn_3_O_4_) when heated, that can be shown by the next schemes:

4 $$ 4\ \mathrm{SnO}\to \mathrm{S}{\mathrm{n}}_3{\mathrm{O}}_4+\mathrm{Sn} $$

5 $$ 3\ \mathrm{SnO}\to \mathrm{S}{\mathrm{n}}_2{\mathrm{O}}_3+\mathrm{Sn} $$

In turn, the oxidation of metallic tin by oxygen of air is accompanied with generation of SnO and following repeating of reaction (4 and 5). Analysis of literature revealed that the rate of formation of mixture oxides (Schemes 4–5) and their disproportioning depends on many factors: composition of the initial precursors and conditions of reaction for fabrication of SnO and following thermal annealing regime. As it was shown that the complete oxidation to SnO_2_ is usually observed at annealing temperatures over 450 °C.

**Fig. 4:**
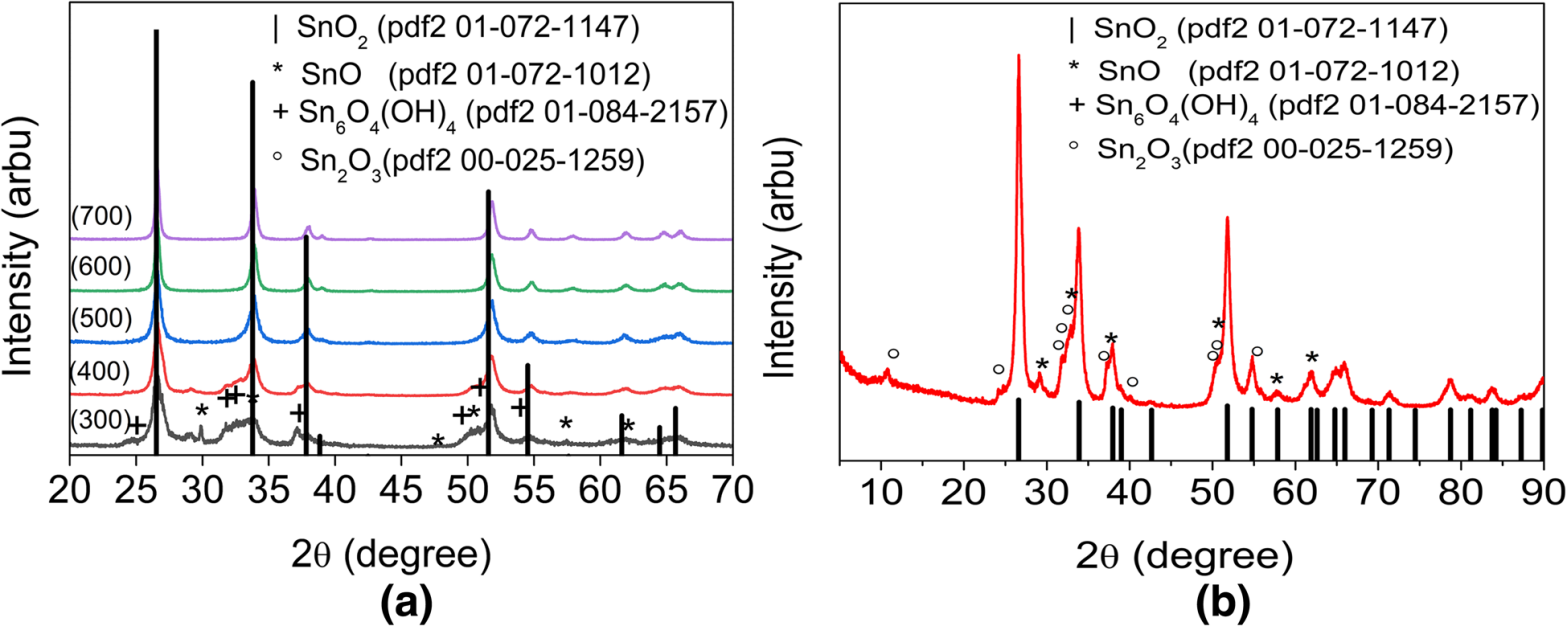
**a** The XRD patterns of the HTONF annealed at different temperatures: 300 °C; 400 °C; 500 °C; 600 °C; 700 °C. **b** The XRD pattern of the HTONF annealed at 400 °C (step 0.02°, integration time 5 s/point)

From the results of XRD data (Fig. 4a), for the samples annealed at 300 °C, the three types of phases were observed: SnO_2_, Sn_6_O_4_(OH)_4_, SnO. Respectively, the simultaneous dehydration of Sn_6_O_4_(OH)_4_ and oxidation to SnO_2_ has place. In the XRD of samples annealed at 400 °C, the peaks of the phase Sn_6_O_4_(OH)_4_ are already absent that is in well agreement with the results of the thermogravimetric analysis (Fig. 3). At the same time, the peaks of low intensity that can be attributed to the phases SnO and Sn_2_O_3_ are observed (400 °C). In order to specify the composition of the material, we made a detailed XRD analysis (Fig. 4b). It allowed to confirm the previous analysis and shows phases SnO [17, 20–29] and Sn_2_O_3_ [22] that were also observed in other studies. Increasing the annealing temperature to 500 °C leads to the complete oxidation of the phases consisting of Sn (II) and formation of pure SnO_2_. Further increasing of the temperature (600–700 °C) does not result in sufficient changes of phase composition of the samples.

**Fig. 5:**
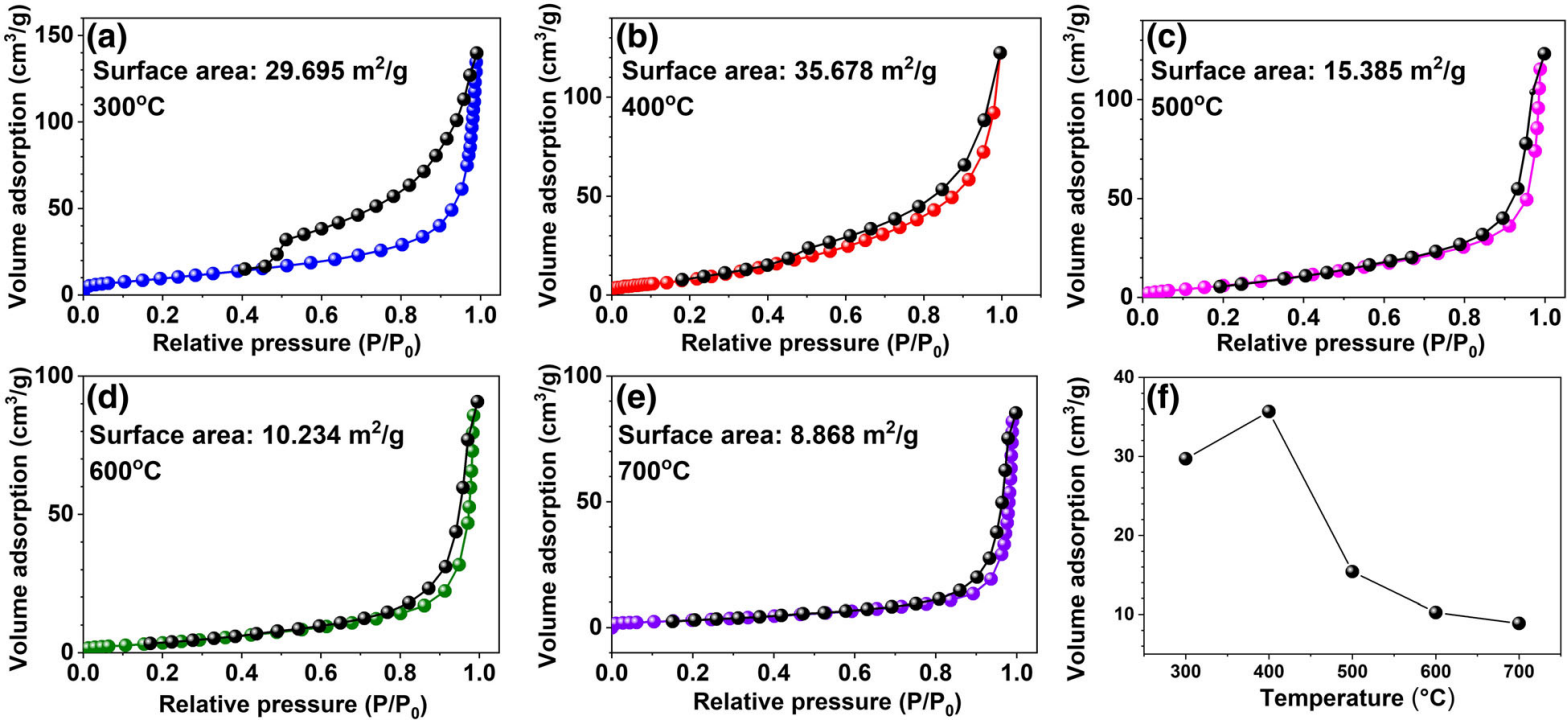
**a**–**e** The nitrogen absorption-desorption isotherms of HTONF annealed in air at different temperatures: 300 °C, 400 °C, 500 °C, 600 °C, 700 °C. The black curve presents absorption and color curves present desorption processes. **f** The curve of the relationship of annealing temperatures and specific surface area

The nitrogen absorption-desorption isotherms and pores size distribution curve are shown in Fig. 5. From Fig. 5a–e, it is clearly seen that the as-synthesized HTONF samples annealed at 400 °C show the biggest surface area (35.678 m^2^/g), which provides the biggest number of active sites and improves the gas sensitivity performance of the HTONF [30, 31]. In our opinion, the changes in specific surface area are part of a complex process of phase transformation accompanied with growth of nanostructures of different shapes such as leaves, needles, membranes, etc. [32–35] that strongly depends on conditions of synthesis process.

### SEM and TEM Analysis

**Fig. 6:**
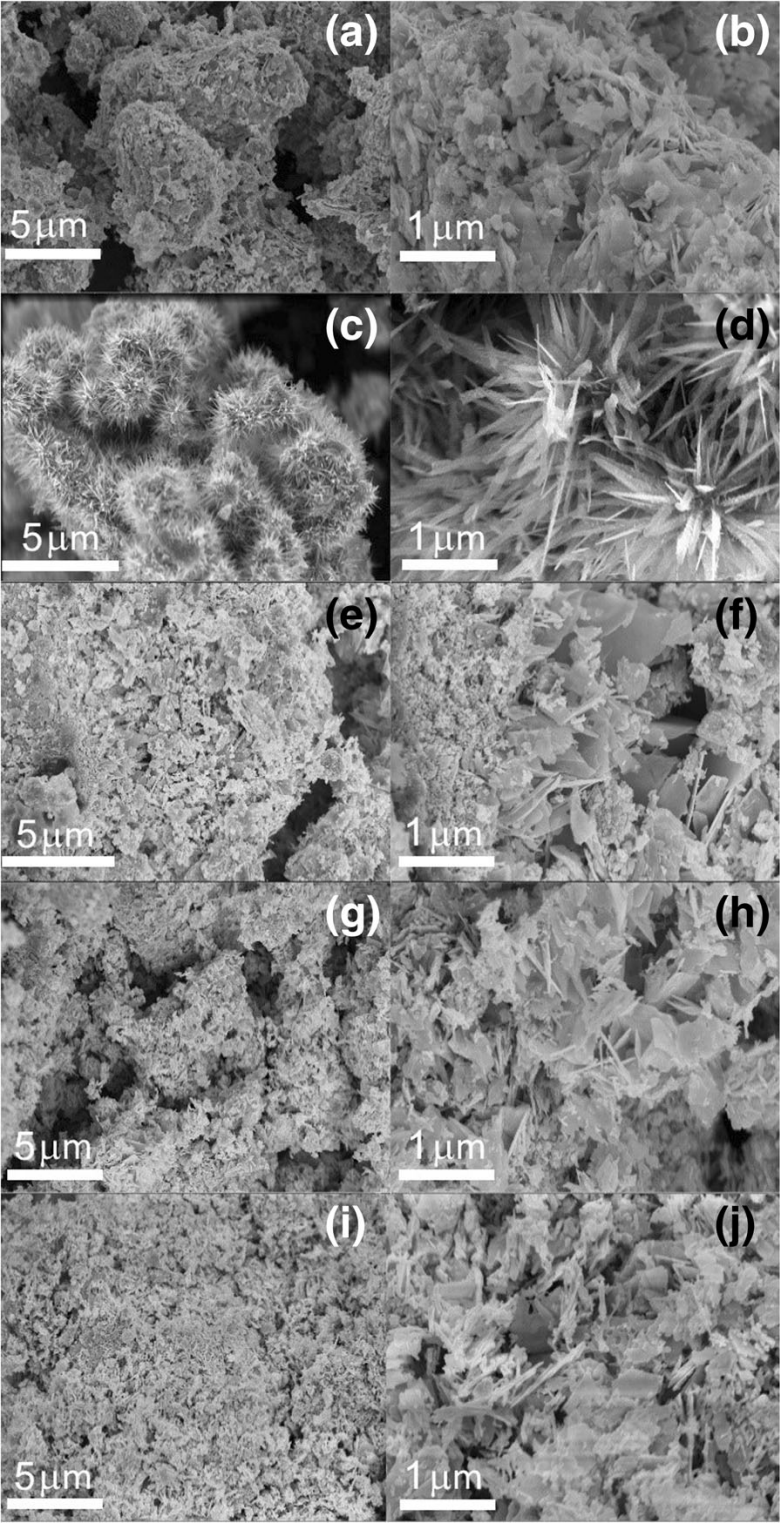
SEM images of the HTONF annealed at 300 °C (**a**, **b**); 400 °C (**c**, **d**); 500 °C (**e**, **f**); 600 °C (**g**, **h**); 700 °C (**i**, **j**)

The morphology of the as-synthesized HTONF was investigated by scanning electron microscopy (SEM), as illustrated in Fig. 6. Figure 6c presents the SEM picture of the sample that forms flower-like morphology and shows good uniformity. Meanwhile, no other morphologies are observed in Fig. 6c, indicating that the proposed experimental procedure leads to formation of the only hierarchical nanoflower morphology of the product [36, 37]. A SEM image of an individual HTONF is shown in Fig. 6d. One can see that the HTONF is assembled with a lot of acicular-like nanosheets forming a shape of *Callistephus chinensis* flower.

It should be noted that the temperature of the hydrothermal synthesis sufficiently influenced on the morphology of the obtained composites. And SEM image of as-obtained HTONF annealed at different temperatures were shown in Fig. 6a–j. From this picture, it is very clearly seen that the morphologies of tin oxide materials will change when the annealing temperature is increased, and the tin oxide materials annealed at 400 °C shows the morphology similar to magnificent fractal structures. Such morphology should have specific surface area higher than other sintered disordered powder-like materials and it was confirmed by BET measurements (Fig. 5). As it was noted in [23], the as-obtained composite forms cubic shape with surface covered by nano-sheets at the temperature 140 °C. Thus, the temperature is the factor that obviously effects on the nucleation process and following growth of tin-containing composites. And the morphology of as-prepared materials will be changed when the calcination temperature changes.

**Fig. 7:**
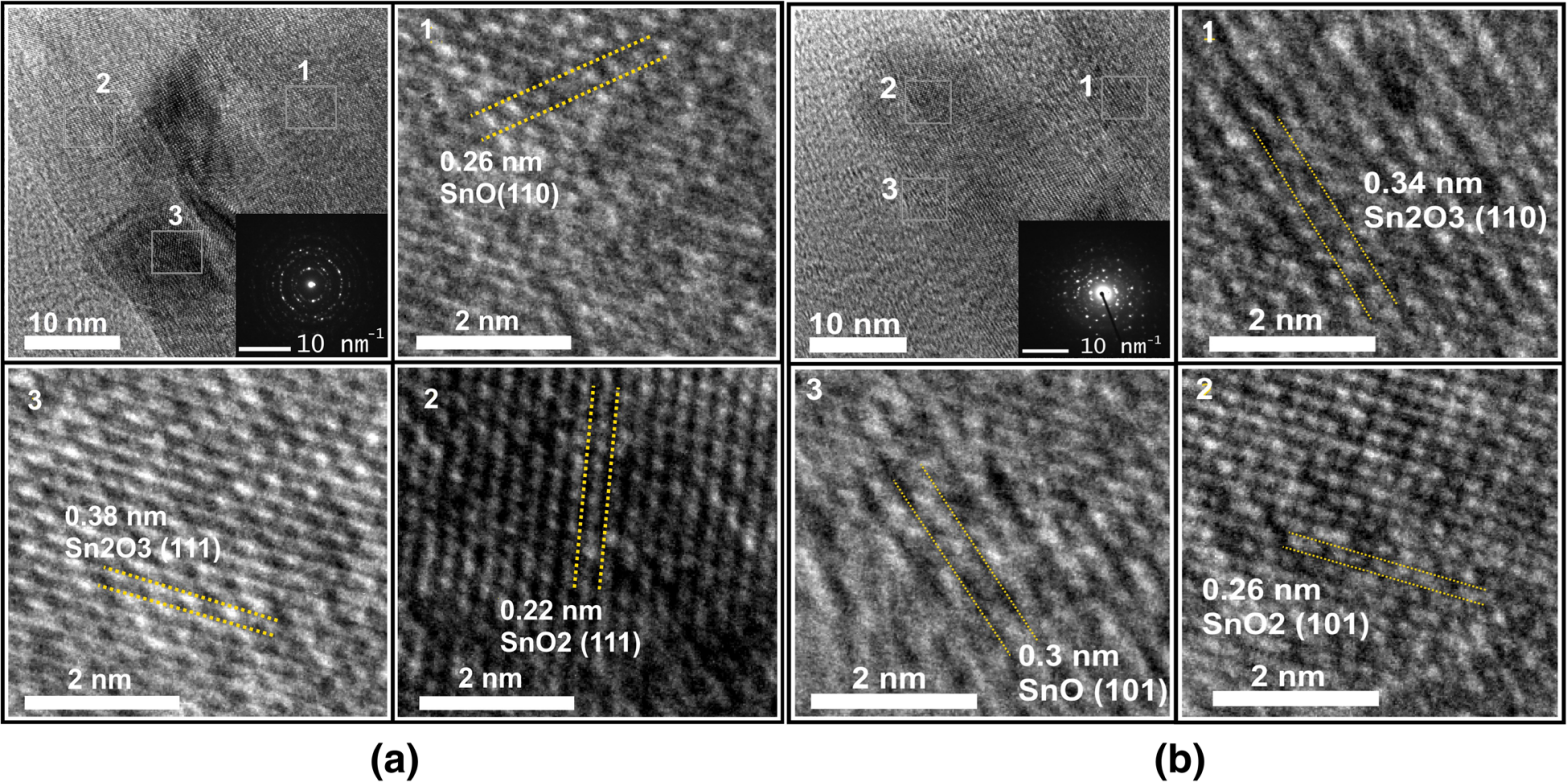
HRTEM images and corresponding SAED patterns of HTONF annealed at 300 °C and 400 °C, respectively. **a** SnO_2_—300 °C; **b** SnO_2_—400 °C

The as-obtained HTONF were further investigated by HRTEM. The HRTEM images and corresponding SAED patterns of HTONF annealed at 300 °C and 400 °C are shown in Fig. 7a, b. From these pictures, it clearly indicated that as-synthesized HTONF samples annealed at 300 °C and 400 °C have polycrystalline structure and consist of SnO_2_, SnO, and Sn_2_O_3_ phases which is in agreement with the above XRD results and results of other research teams [35, 38].

### Gas Sensing Properties

**Fig. 8:**
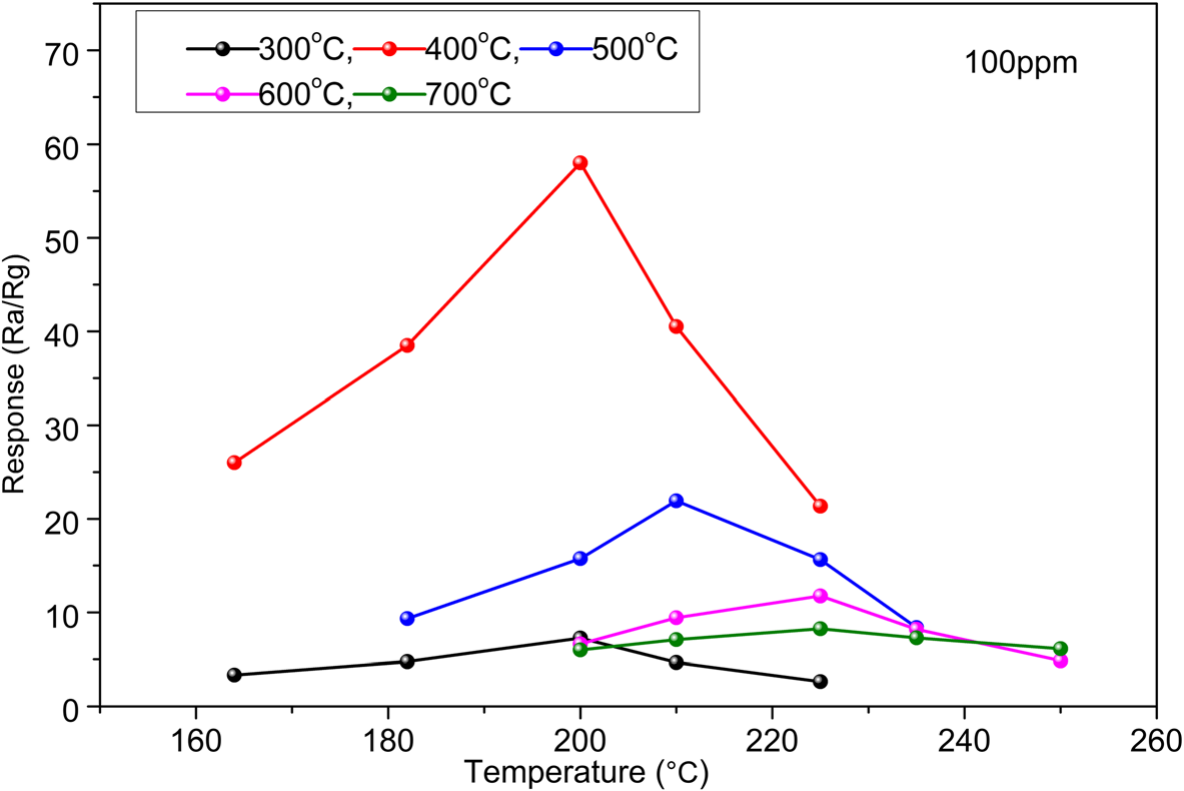
The relationship of response to 100 ppm methanol versus the operating temperature of gas sensor based on the as-prepared sample annealed at different temperatures

It is well known that gas sensing properties of metal oxide semiconductor gas sensors are highly dependent on the operating temperatures. In order to find out the optimal operating and annealing temperature, the HTONF gas sensors were investigated for different temperatures. Figure 8 shows the sensitivity of the as-synthesized HTONF gas sensor to 100 ppm methanol as a function of the operating temperature ranging from 164 to 265 °C. It can be seen that the as-prepared gas sensor based on the sample annealed at 400 °C exhibits the maximum response of 58 at 200 °C. The gas sensitivity greatly rises and reaches a maximum response when the operating temperature increases up to 200 °C, and then the gas sensitivity drastically decreases at further rising of the operating temperature. The relationship of sensitivity and operating temperatures can be explained by mechanism of gas adsorption and desorption on the surface of semiconductor metal oxide gas sensor [39]. The chemical activity of a gas sensor is rather weak at low operating temperature, which leads to low sensitivity. The desorption rate of gas increases with growing the sensor surface temperature, and at a certain temperature it will exceed the adsorption rate that results in sensitivity drop [40]. Therefore, 200 °C and 400 °C were defined experimentally as the optimum operating temperature for the gas sensing measurement and calcination temperature, respectively.

Table 1 indicates that the HTONF gas sensor possesses a better gas sensitivity to methanol at low working temperature compared with sensors based on other complicated structures. So, the studied hierarchical nanoflower structure would sufficiently improve the gas sensing performance of tin oxide material that can be used for fabrication of high-sensitivity low-cost methanol sensors.

**Table 1:** Comparison of methanol gas sensors based on tin oxide materials with different morphologies

Gas sensor	Operating temperature, °C	Methanol concentration, ppm	Sensitivity value	Reference
HTONF	200	100	58	This work
SnO_2_ hierarchical porous nanosheets	275	100	3	[32]
Rolled-up SnO_2_ nanomembranes	220	10	2	[33]
Hollow SnO_2_ microfiber	270	100	10	[34]
Honeycomb-like SnO_2_	320	100	18	[35]

**Fig. 9:**
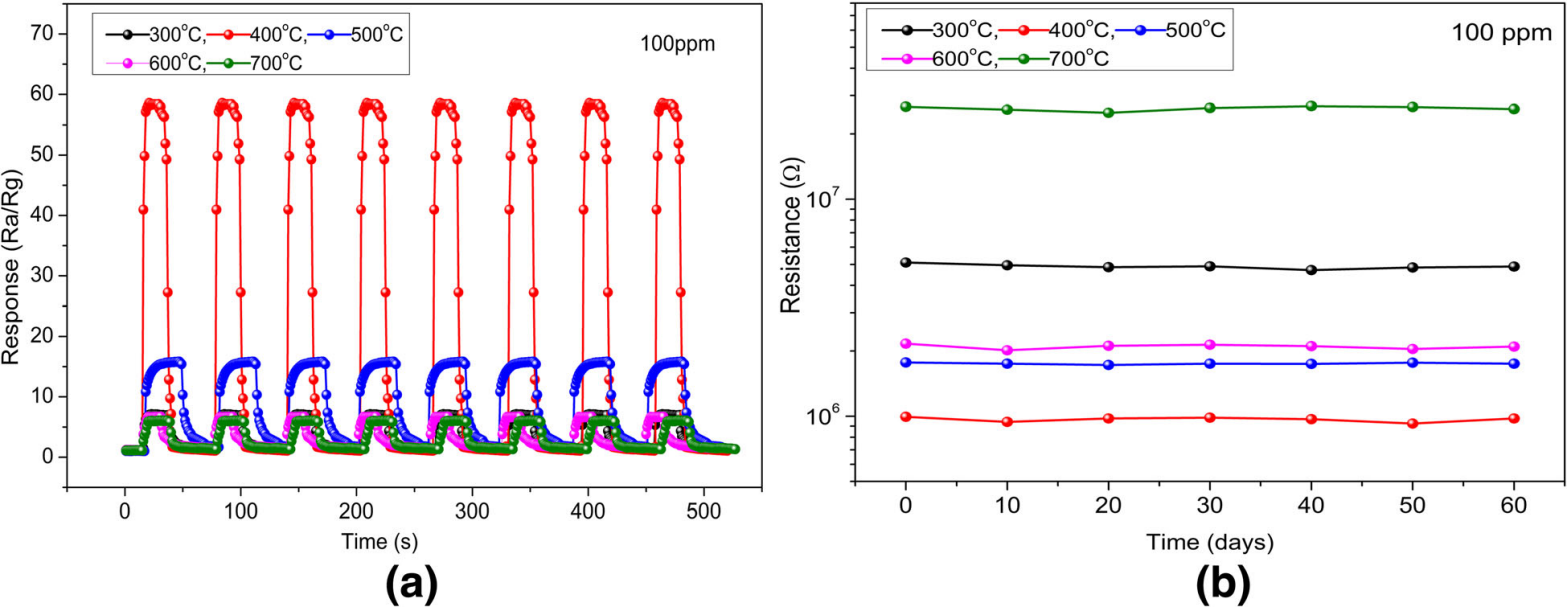
**a** Dynamic response of gas sensor based on HTONF annealed at different temperatures during eight cycles of exposure to 100 ppm methanol at 200 °C. **b** The long-term initial resistance stability of HTONF sensor annealed at different temperatures to 100 ppm methanol in dry air at 200 °C

To further analyze the reproducibility and long-term stability of gas sensor, the HTONF gas sensors with calcination at different temperatures were tested by carrying out eight cycles of response measurements of 100 ppm methanol, as-fabricated, and 60 days aged. As shown in Fig. 9a, the gas sensor annealed at the 400 °C presents good sensitivity and repeatability without visible changes after eight-cycle examination. The response and recovery time to 100 ppm methanol at 200 °C were about 4 s and 8 s, respectively. The gas sensibility was about 58 which means that the fabricated gas sensor has high sensitivity and repeatability. And this may be attributed to the hierarchical structure suitable for the pervasion and detecting methanol gas [41, 42]. From Fig. 9b, it is fairly observed that the as-synthesized gas sensor annealed at 400 °C shows good stability at the operating temperature of 200 °C within 60 days. The above results indicate that the HTONF gas sensor can be a good methanol detector [43, 44].

**Fig. 10:**
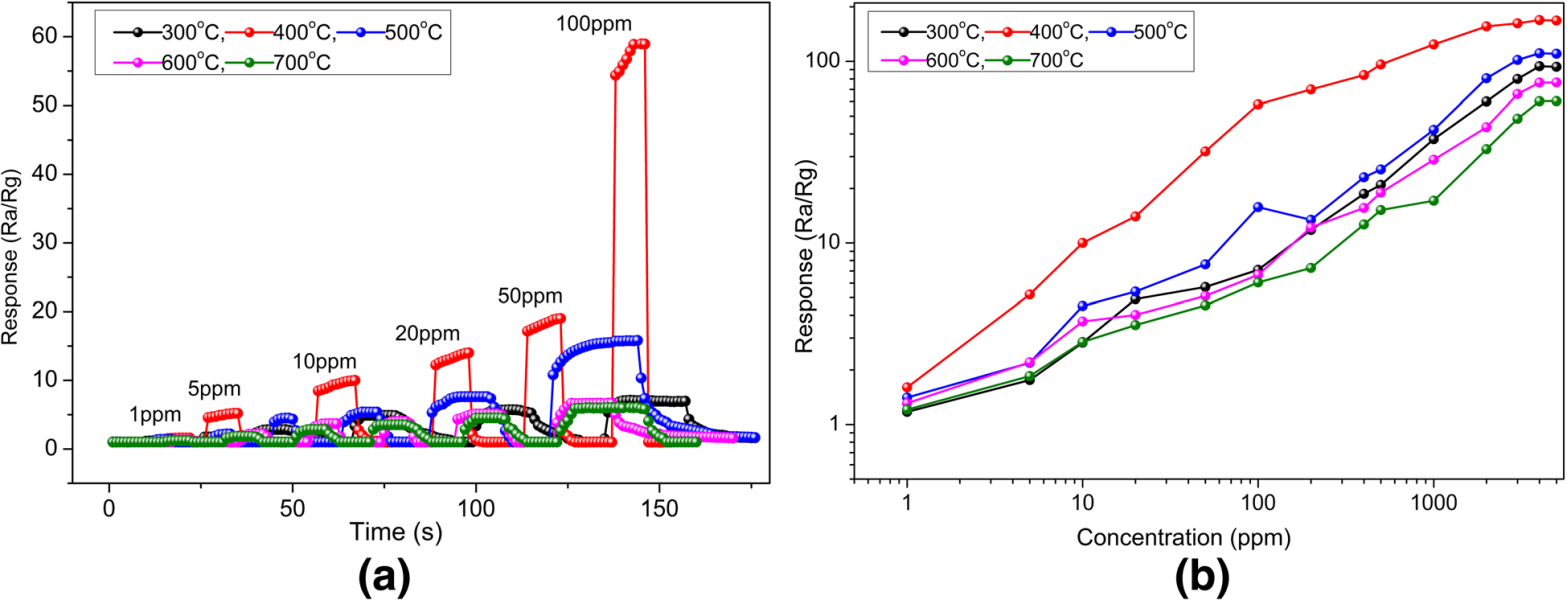
**a** Typical response and recovery curves of HTONF annealed at different temperatures to different concentrations of methanol gas at optimum operating temperature. **b** Response of HTONF annealed at different temperatures to various methanol concentrations at the optimum operating temperature

Figure 10a displays the dynamic response and recovery curves of the as-obtained sensor for different concentrations of methanol molecules ranging from 1 to 100 ppm at the 200 °C. It is obvious that the HTONF gas sensor annealed at 400 °C shows the good response and recovery performance. Moreover, the fabricated gas sensor has shown the response of 1.6 for the concentration of methanol as low as 1 ppm, demonstrating that the HTONF sensor can detect extremely low concentrations of methanol [45, 46].

In addition, the response behavior of the gas sensor to different concentrations of methanol at 200 °C is shown in Fig. 10b. It can be easily found that the sensor presents upward tendency when the concentration of methanol is increased. The gas sensitivity depends linearly on the concentrations of methanol vapor varying from 1 to 100 ppm. However, the gas sensitivity saturates when the concentration of methanol exceeds 2000 ppm. The phenomenon can be explained as follows: the methanol molecules are absorbed on the surface of the HTONF and participate in surface reaction, resulting in the rise of gas sensing. The gas sensor is saturated when the concentration of methanol exceeds a threshold value, and the gas sensitivity of the sensor shows slow growth [47, 48].

**Fig. 11:**
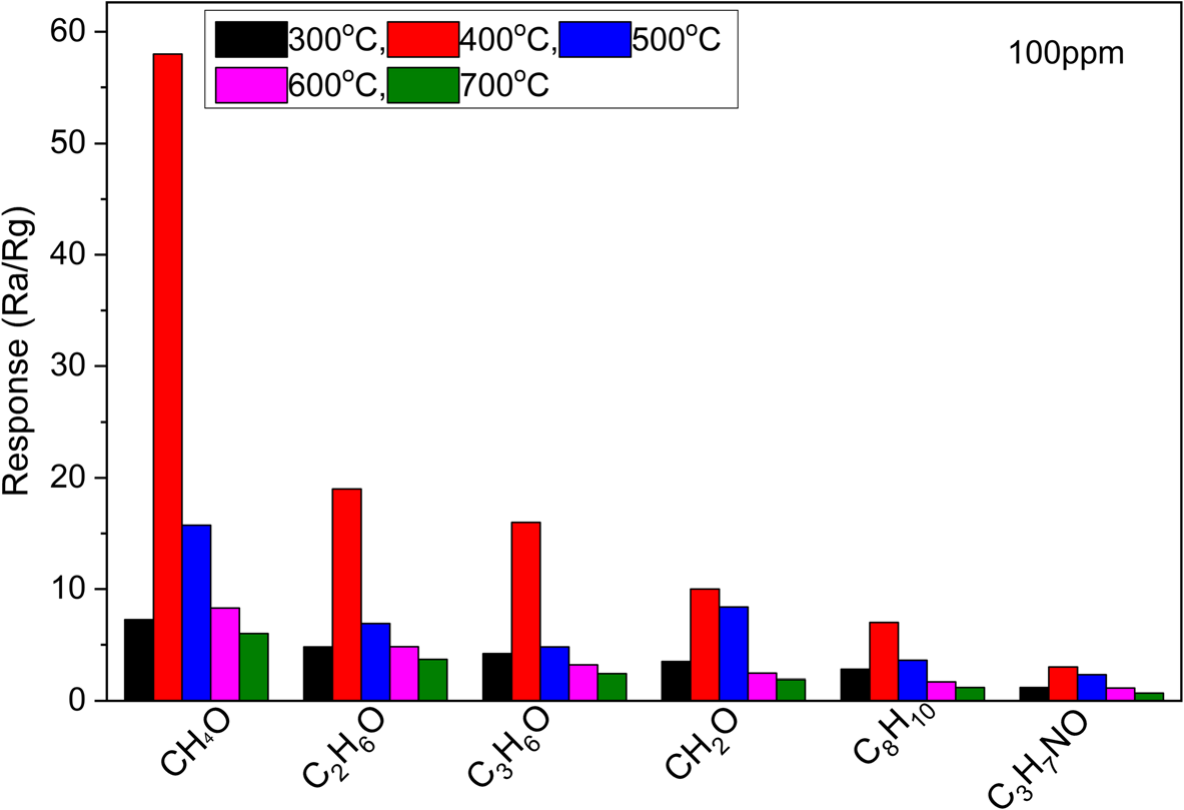
The response of HTONF annealed at different temperatures to 100 ppm methanol, ethanol, acetone, formaldehyde, paraxylene, and dimethylformamide at the operating temperature of 200 °C

The sensor selectivity is a kind of extremely important parameter for sensors in the wide practical applications. The fabricated sensor has been additionally analyzed in terms of the selectivity. Figure 11 shows the gas sensing performance of the HTONF sensor to 100 ppm methanol, ethanol, acetone, formaldehyde, paraxylene, and dimethylformamide at 200 °C. Different gases have different chemical properties, leading to different gas sensitivities of the fabricated gas sensor [49, 50]. It is clearly seen that the gas sensitivity of the HTONF gas sensor annealed at 400 °C to 100 ppm methanol is 58 while the responses to 100 ppm ethanol, acetone, formaldehyde, paraxylene, and dimethylformamide are 19, 16, 10, 7, and 3, respectively. It is obviously seen that the HTONF gas sensor is much more sensitive to methanol compared with other studied gases demonstrating a high selectivity to methanol.

### Gas Sensing Mechanism

**Fig. 12:**
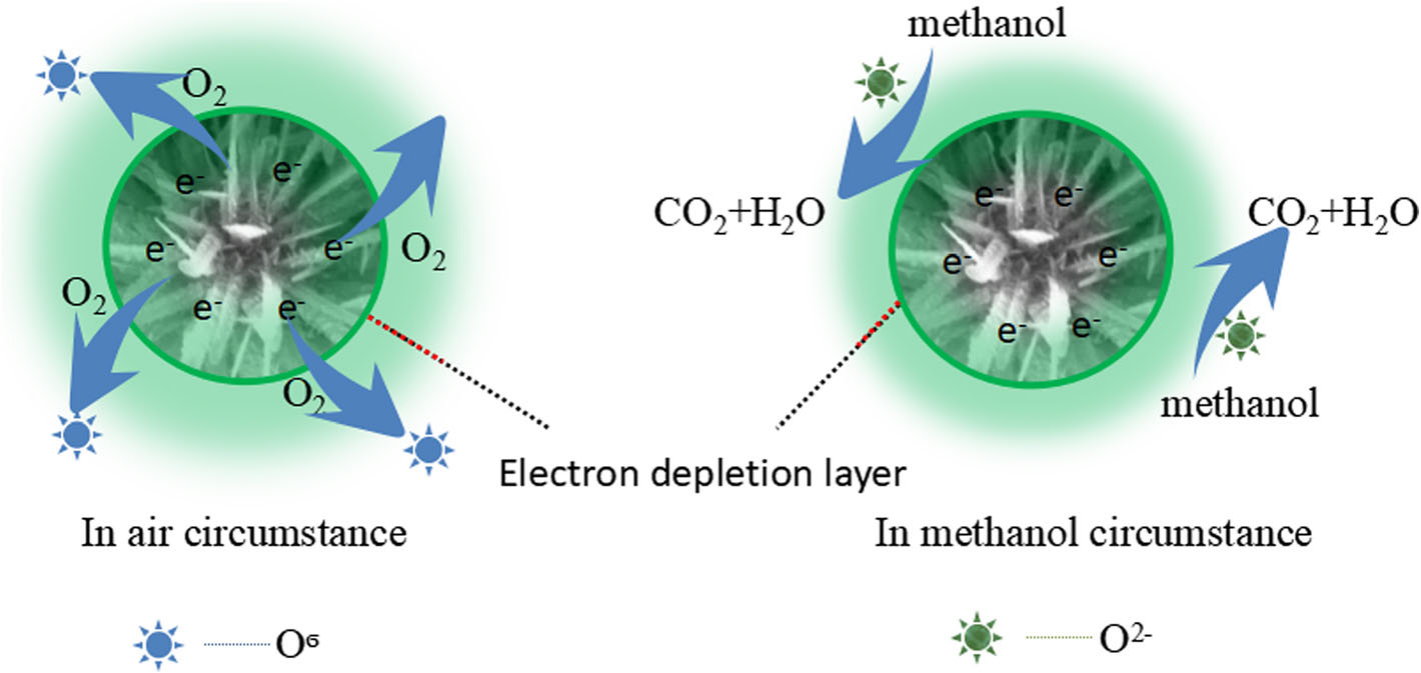
The diagram of gas sensing mechanism of HTONF

The sensing mechanism of metal oxide gas sensors has been researched and reported in previous works [51, 52]. It was stated that gas sensor response mechanism mainly refers to the reaction between the target gas and the surface chemisorbed oxygen ions, which leads to changes in the resistance of the gas sensor. In order to understand the gas sensing mechanism for the as-obtained HTONF gas sensor, a schematic diagram of the mechanism is shown in Fig. 12. Generally, the reaction of gas sensor based on the as-prepared sample can be divided into two parts: firstly, when the HTONF are exposed to the air, oxygen molecules in air are absorbed on the surface of HTONF and create a number of chemisorbed oxygen species (O_2_(ads)^−^, O(ads)^−^, and O(ads)^2−^) by capturing free electrons in conduction band, which could extremely decrease the density of free carriers and form an electron depletion layer on the surface of HTONF. Therefore, the resistance of HTONF in air will increase (*R*_*a*_) [43, 44]. The process can be explained as follows:

6 $$ {\mathrm{O}}_2\left(\mathrm{gas}\right)\to {\mathrm{O}}_2\left(\mathrm{ads}\right) $$

7 $$ {\mathrm{O}}_2\left(\mathrm{ads}\right)+{\mathrm{e}}^{-}\to {\mathrm{O}}_2^{-}\left(\mathrm{ads}\right) $$

8 $$ {\mathrm{O}}_2^{-}\left(\mathrm{ads}\right)+{\mathrm{e}}^{-}\to 2{\mathrm{O}}^{-}\left(\mathrm{ads}\right) $$

9 $$ 2{\mathrm{O}}^{-}\left(\mathrm{ads}\right)+{\mathrm{e}}^{-}\to {\mathrm{O}}^{2-}\left(\mathrm{ads}\right) $$

Secondly, when the as-obtained HTONF gas sensor is exposed to methanol gas, the chemisorbed oxygen species react with methanol and release electrons back to the conduction band. So, the electron depletion layer is reduced and the surface resistance (*R*_*g*_) of HTONF is decreased. The chemical reaction can be presented as follows:

10 $$ \mathrm{C}{\mathrm{H}}_3\mathrm{OH}\left(\mathrm{gas}\right)\to {\mathrm{CH}}_3\mathrm{OH}\ \left(\mathrm{ads}\right) $$

11 $$ {\mathrm{CH}}_3\mathrm{OH}\ \left(\mathrm{ads}\right)+3\ {\mathrm{O}}^{2-}\left(\mathrm{ads}\right)\to {\mathrm{CO}}_2+2\ {\mathrm{H}}_2\mathrm{O}+6\ {\mathrm{e}}^{-} $$

According to the Knudsen approach to diffusion in pores, the gas diffusivity is:

12 $$ {D}_k=\frac{3r}{4}\ \sqrt{\frac{2 RT}{\pi M}} $$

where *r*, *T*, and *M* are pore diameter, operating temperature, and molecular mass, respectively. From this formula, it can be easily concluded that higher operating temperature, bigger pores diameter, and lighter molecular mass play an important role in increasing the diffusion rate of detected gas [53, 54]. Therefore, the remarkable gas sensing properties of HTONF to methanol could be caused by the hierarchical structure. The hierarchical structure has a large surface area contributing to gas adsorption and desorption. As a result, the as-obtained HTONF gas sensor exhibits high sensitivity, quick response and recovery rates, and selectivity.

The presence of humidity in the atmosphere should decrease the methanol gas sensing performance of sensor by increasing the conductivity of the metal oxide gas sensor as it was described in [55]. It is noticeable that the sensing behavior of the HTONF sensor to formaldehyde having molecular mass less than methanol, the lower sensitivity was observed comparing to methanol. The methanol and other alcohols contain hydroxyl group that allows its easy adsorption on surface of HTONF with chemisorbed oxygen. At the same time, the least molecular mass of the methanol in comparison with other alcohols provides its fast diffusion in pores of the fabricated HTONF increasing sensitivity of the material in accordance with the Knudsen approach (Eq. 12). In the series of studied gases, the noticeable is low sensitivity of the fabricated sensor to formaldehyde (Fig. 11). Although the formaldehyde molecule is even less than methanol one, it contains highly negative-polarized O atom that sufficiently impedes absorption of the molecule on the surface of material with chemisorbed oxygen species and consequently sensitivity of the device to that gas is low.

In light of the results obtained from XRD spectra, thermogravimetric curve, and surface area measurements, the model of sensitivity we proposed also accounts the next two reasons. The material annealed at temperatures below 450 °C includes several phases of tin oxides: SnO_2_, Sn_2_O_3_, and Sn_3_O_4_. These phases are responsible, by our opinion, for high sensitivity properties of the obtained structures, as it was reported by different research teams in recent publications.

The second factor is the high specific surface area of the sample 400 °C. Annealing of the samples at temperature 300 °C and 500 °C and higher results in formation of the developed surface with area value less than that of the sample annealed at 400 °C. The two described factors effect onto the sensitivity in opposite ways. And the compromise annealing temperature 400 °C resulted in simultaneously high value of the surface area and the high-sensitive tin oxides Sn_2_O_3_ and Sn_3_O_4_. It led to the highest sensitivity of the fabricated sensor based on HTONF.

## Conclusions

In summary, hierarchical tin oxide nanoflowers for gas sensors have been synthesized via one-type hydrothermal route. A series of results indicates that the HTONF gas sensor shows high gas sensitivity, short response and recovery time, long stability, and high reproducibility. The gas response of the as-obtained gas sensor to 100 ppm methanol is about 58 and response/recovery time is about 4 s and 8 s, respectively. The excellent gas sensing performance of the HTONF sensor is attributed to the original hierarchical structure and phase composition of tin oxides. The HTONF could be a desirable candidate for applications in sensor area.

## Abbreviations

BET: Brunauer-Emmett-Teller
HTONF: Hierarchical tin oxide nanoflowers
SEM: Scanning electron microscopy
TEM: Transmission electron microscopy
TGA: Thermal gravimetric analysis
XRD: X-ray powder diffraction Instrument

## References

[1] Das S, Murthy ASR, Gnanasekar KI, Jayaraman V (2018). Solution processed Mg^+2^:LaNiO_3_ thin films for effective methanol sensing. Sens Actuator B.

[2] Ji HF, Liu WK, Li S, Li Y, Shi ZF, Tian YT, Li XJ (2017). High-performance methanol sensor based on GaN nanostructures grown on silicon nanoporous pillar array. Sens Actuator B.

[3] Wang LL, Li ZJ, Luo L, Zhao CZ, Kang LP, Liu DW (2016). Methanol sensing properties of honeycomb-like SnO_2_ grown on silicon nanoporous pillar array. J Alloys Compd.

[4] Quy CT, Thai NX, Hoa ND, Le DTT, Hung CM, Duy NV, Hieu NV (2018). C_2_H_5_OH and NO_2_ sensing properties of ZnO nanostructures: correlation between crystal size defect level and sensing performance. RSC Adv.

[5] Tan WH, Tan JF, Li L, Dun MH, Huang XT (2017). Nanosheets-assembled hollowed-out hierarchical Co_3_O_4_ microrods for fast response/recovery gas sensor. Sens Actuator B.

[6] Bulemo PM, Cho HJ, Kim DH, Kim ID (2018). Facile synthesis of Pt-functionalized Meso/macroporous SnO_2_ hollow spheres through in situ templating with SiO_2_ for H_2_S sensors. ACS Appl Mater Interfaces.

[7] Rong Q, Zhang YM, Wang C, Zhu ZQ, Zhang J, Liu QJ (2017). A high selective methanol gas sensor based on molecular imprinted Ag-LaFeO_3_ fibers. Sci Rep.

[8] Chen GH, Ji SZ, Li HD, Kang XL, Chang SJ, Wang YN, Yu GW, Lu JR (2015). High-energy faceted SnO (2)-coated TiO (2) Nanobelt Heterostructure for near-ambient temperature-responsive ethanol sensor. ACS Appl Mater Interfaces.

[9] Wang QJ, Kou XY, Liu C, Zhao LJ, Lin TT, Liu FM (2018). Hydrothermal synthesis of hierarchical CoO/SnO_2_ nanostructures for ethanol gas sensor. J Colloid Interface Sci.

[10] Shu SM, Wang MX, Yang W, Liu ST (2017). Synthesis of surface layered hierarchical octahedral-like structured Zn _2_ SnO_4_/SnO_2_ with excellent sensing properties toward HCHO. Sens Actuator B.

[11] Zhang R, Zhou TT, Wang LL, Zhang T (2018). Metal-organic frameworks-derived hierarchical Co_3_O_4_ structures as efficient sensing materials for acetone detection. ACS Appl Mater Interfaces.

[12] Han D, Song P, Zhang S, Zhang HH, Xu Q, Wang Q (2015). Enhanced methanol gas-sensing performance of Ce-doped In_2_O_3_ porous nanospheres prepared by hydrothermal method. Sens Actuator B.

[13] Qin J, Cui ZD, Yang XJ, Zhu SL, Li ZY, Liang YQ (2015). Synthesis of three-dimensionally ordered macroporous LaFeO_3_ with enhanced methanol gas sensing properties. Sens Actuator B.

[14] Khardani M, Bouaïcha M, Boujmil MF, Bessaïs B (2010). Aluminum-mesoporous silicon coplanar type structure for methanol gas sensing. Micropor Mesopor Mater.

[15] Ji FX, Ren XP, Zheng XY, Liu YC, Pang LQ, Jiang JX, Liu SZ (2016). 2D-MoO_3_ nanosheets for superior gas sensors. Nanoscale.

[16] Zhang ZY, Zou XM, Xu L, Liao L, Liu W, Ho J, Xiao X, Jiang CZ, Li J (2015). Hydrogen gas sensor based on metal oxide nanoparticles decorated graphene transistor. Nanoscale.

[17] Feng LD, Huang XJ, Choi YK (2006). Dynamic determination of domestic liquefied petroleum gas down to several ppm levels using a Sr-doped SnO_2_ thick film gas sensor. Microchim Acta.

[18] Mali SS, Patil JV, Kim H, Hong CK (2018). Synthesis of SnO_2_ nanofibers and nanobelts electron transporting layer for efficient perovskite solar cells. Nanoscale.

[19] Holleman AF, Wiberg E, Tin FA (2001) Holleman - Wiberg Inorganic Chemistry. Academic Press, New-York. p. 904

[20] Yu H, Yang TY, Wang ZY, Li ZF, Xiao BX, Zhao Q, Zhang M (2017). Facile synthesis cedar-like SnO_2_ hierarchical micro-nanostructures with improved formaldehyde gas sensing characteristics. J Alloys Compd.

[21] Yang DJ, Kamienchick I, Youn DY, Rothschild A, Kim ID (2010). Ultrasensitive and highly selective gas sensors based on electrospun SnO_2_ nanofibers modified by Pd loading. Adv Funct Mater.

[22] Murken VG, Tromel M (1973). Uber das bei der Disproportionierung von SnO entstehende Zinnoxid, Sn_2_0_3_. Z anorg. Allg Chem.

[23] Yu H, Yang TY, Wang ZY, Li ZF, Zhao Q, Zhang MZ (2018). p-N heterostructural sensor with SnO-SnO_2_ for fast NO_2_ sensing response properties at room temperature. Sens Actuator B.

[24] Zhang YQ, Li D, Qin LG, Zhao PL, Liu FM, Chuai XH, Sun P (2018). Preparation and gas sensing properties of hierarchical leaf-like SnO_2_ materials. Sens Actuator B.

[25] Wang Q, Yao N, An DM, Li Y, Zou YL, Lian XX, Tong XQ (2016). Enhanced gas sensing properties of hierarchical SnO_2_ nanoflower assembled from nanorods via a one-pot template- free hydrothermal method. Ceram Int.

[26] Suman PH, Longo E, Orlandi MO (2014). Controlled synthesis of layered Sn_3_O_4_ nanobelts by carbothermal reduction method and their gas sensor properties. J Nanosci Nanotechnol.

[27] He YH, Li DZ, Chen J, Shao Y, Xian JJ, Zheng XZ, Wang P (2014). Sn_3_O_4_: A novel heterovalent-tin photocatalyst with hierarchical 3D nanostructures under visible light. RSC Adv.

[28] Balgude SD, Sethi YA, Kale BB, Munirathnam NR, Amalnerkar DP, Adhyapak PV, He Y (2014). Sn_3_O_4_: a novel heterovalent-tin photocatalyst with hierarchical 3D nanostructures under visible light. RSC Adv.

[29] Patil JV, Kim H, Hong CK (2018). Synthesis of SnO_2_ nanofibers and nanobelts electron transporting layer for efficient perovskite solar cells. Nanoscale.

[30] Stanoiu A, Simion CE, Sackmann A, Baibarac M, Florea OG (2018). Networked mesoporous SnO_2_ nanostructures templated by Brij ® 35 with enhanced H_2_S selective performance. Micropor Mesopor Mater.

[31] Yang FC, Guo ZG (2016). Tuning SnO_2_ architectures with unitary or composite microstructure for the application of gas sensors. J Colloid Interface Sci.

[32] Zhao CH, Gong HM, Lan WZ, Xu H, Liu S, Wang F (2018). Facile synthesis of SnO_2_ hierarchical porous nanosheets from graphene oxide sacrificial scaffolds for high-performance gas sensor. Sens Actuator B.

[33] Liu XH, Ma TT, Xu YS, Sun L, Zheng LL, Schmidt OG, Zhang J (2018). Rolled-up SnO_2_ nanomembranes: a new platform for efficient gas sensors. Sens Actuator B.

[34] Zou YH, Chen S, Sun J, Liu JQ, Che YK, Liu XH, Zhang J, Yang DJ (2017). Highly efficient gas sensor using a hollow SnO_2_ microfiber for triethylamine detection. ACS Sens.

[35] L XY, Peng K, Dou YW, Chen JS, Zhang Y, An G (2018) Facile synthesis of wormhole-like mesoporous tin oxide via evaporation induced self-assembly and the enhanced gas-sensing properties. Nanoscale Res Lett 13:14 10.1186/s11671-018-2434-4 PMC5764904 29327243

[36] Hu J, Wang T, Wang YJ, Huang D, He GL, Han YT, Hu NT (2018). Enhanced formaldehyde detection based on Ni doping of SnO_2_ nanoparticles by one-step synthesis. Sens Actuator B.

[37] Tammanoon N, Wisitsoraat A, Phokharatkul D, Tuantranont A, Phanichphant S, Yordsri V, Liewhiran C (2018). Highly sensitive acetone sensors based on flame-spray-made La_2_O_3_ -doped SnO_2_ nanoparticulate thick films. Sens Actuator B.

[38] Cheng DL, Hou XX, Wen HJ, Wang Y, Wang HL, L XJ, Lu HX (2010). The enhanced alcohol-sensing response ultrathin WO3 nanoplates. Nanotechnology.

[39] Ma YH, Lu Y, Gou HT, Zhang WX, Yan SH, Xu XL (2018). Octahedral NiFe_2_O_4_ for high-performance gas sensor with low working temperature. Ceram Int.

[40] Xu SP, Zhao HP, Xu Y, Xu R, Lei Y (2018). Carrier mobility-dominated gas sensing: a room-temperature gas-sensing mode for SnO_2_ nanorod array sensors. ACS Appl Mater Interfaces.

[41] Tan JF, Dun MH, Li L, Zhao JY, Tan WH, Lin ZD, Huang XT (2017). Synthesis of hollow and hollowed-out Co_3_O_4_ microspheres assembled by porous ultrathin nanosheets for ethanol gas sensors: responding and recovering in one second. Sens Actuator B.

[42] Xiong Y, Zhu ZY, Ding DG, Lu WB, Xue QZ (2018). Multi-shelled ZnCo_2_O_4_ yolk-shell spheres for high-performance acetone gas sensor. Appl Surf Sci.

[43] Ma L, Ma SY, Shen XF, Wang TT, Jiang XH, Chen Q, Qiang Z (2018). PrFeO_3_ hollow nanofibers as a highly efficient gas sensor for acetone detection. Sens Actuator B.

[44] Wang JQ, Li ZJ, Zhang S, Yan SN, Cao BB, Wang ZG, Fu YQ (2018). Enhanced NH_3_ gas-sensing performance of silica modified CeO_2_ nanostructure based sensors. Sens Actuator B.

[45] Li YX, Zu BY, Guo YN, Li K, Zeng HB, Dou XC (2016). Surface superoxide complex defects-boosted ultrasensitive ppb-level NO_2_ gas sensors. Small.

[46] Shingange K, Tshabalala ZP, Ntwaeaborwa OM, Motaung DE, Mhlongo GH (2016). Highly selective NH_3_ gas sensor based on au loaded ZnO nanostructures prepared using microwave-assisted method. J Colloid Interface Sci.

[47] Li ZJ, Wang JQ, Zhang S, Yan SN, Cao BB, Shen WZ, Wang ZG, Fu YQ (2018). Highly sensitive NH_3_ gas sensor based on the porous Ce_0.94_Zr_0.06_O_2_ nano-sheets with ppb level detection limit. J Alloys Compd.

[48] Liu X, Chen N, Han BQ, Xiao XC, Chen G, Djerdj I, Wang YD (2015). Nanoparticle cluster gas sensor: Pt activated SnO_2_ nanoparticles for NH3 detection with ultrahigh sensitivity. Nanoscale.

[49] Chen Z, Umar A, Wang SW, Wang Y, Tian T, Shang Y, Fan YZ, Qi Q (2015). Supramolecular fabrication of multilevel graphene-based gas sensors with high NO_2_ sensibility. Nanoscale.

[50] Rai P, Yoon JW, Jeong HM, Hwang SJ, Kwak CH, Lee JH (2014). Design of highly sensitive and selective Au@NiO yolk-shell nanoreactors for gas sensor applications. Nanoscale.

[51] Liu XH, Ma TT, Pinna N, Zhang J (2017). Two-dimensional nanostructured materials for gas sensing. Adv Funct Mater.

[52] Wang Q, Yao N, An DM, Li Y, Zou YL, Lian XX, Tong XQ (2016). Enhanced gas sensing properties of hierarchical SnO_2_ nanoflower assembled from nanorods via a one-pot template-free hydrothermal method. Ceram Int.

[53] Simion CE, Somacescu S, Teodorescu VS, Osiceanu P, Stanoiu A (2018). H_2_S sensing mechanism of SnO_2_-CuWO_4_ operated under pulsed temperature modulation. Sens Actuator B.

[54] Yang HM, Ma SY, Yang GJ, Jin WX, Wang TT, Jiang XH, Li WQ (2016). High sensitive and low concentration detection of methanol by a gas sensor based on one-step synthesis α-Fe_2_O_3_ hollow spheres. Mater Lett.

[55] Kumar A, Kumar M, Kumar R, Singh R, Prasad B, Kumar D (2019). Numerical model for the chemical adsorption of oxygen and reducing gas molecules in presence of humidity on the surface of semiconductor metal oxide for gas sensors applications. Mat Sci Semicon Proc.

